# Correction: *Proceraea exoryxae* sp. nov. (Annelida, Syllidae, Autolytinae), the first known polychaete miner tunneling into the tunic of an ascidian

**DOI:** 10.7717/peerj.3374/correction-1

**Published:** 2018-07-06

**Authors:** Daniel Martin, Arne Nygren, Edwin Cruz-Rivera

**Affiliations:** 1 Centre d’Estudis Avançats de Blanes (CEAB–CSIC), Girona, Spain; 2 Sjöfartsmuseet Akvariet, Göteborg, Sweden; 3 Department of Biological Sciences, University of the Virgin Islands, St. Thomas, Virgin Islands, USA

Corrigendum:

After publication the authors noticed that there are taxonomic and typographical errors in the article. Table 1 has been amended so that Procerastea cornuta Agassiz, 1862 is now Proceraea cornuta (Agassiz, 1862). In the fourth paragraph of the Discussion, “All other species assigned to Proceraea for which stolons are known, i.e., Procerastea cornuta (Agassiz, 1862), Proceraea fasciata Bosc, 1802, P. hanssoni Nygren, 2004, Proceraea nigropunctata Nygren & Gidholm, 2001, P. okadai (Imajima, 1966), and P. prismatica (Müller, 1776), are equipped with achaetous knobs ventral to the first dorsal cirri” is now, “All other species assigned to Proceraea for which stolons are known, i.e., P. cornuta (Agassiz, 1862), P. fasciata Bosc, 1802, P. hanssoni Nygren, 2004, P. nigropunctata Nygren & Gidholm, 2001, P. okadai (Imajima, 1966), and P. prismatica (Müller, 1776), are equipped with achaetous knobs ventral to the first dorsal cirri." The descriptions of (E) and (F) were inverted in the original Figure 5 legend. This has been amended so that legend reads: “(E) Bayonet chaeta and compound chaeta, chaetiger 9 [MNCN 16.01/17717]. (F) Simple and compound chaetae, chaetiger 10 [MNCN 16.01/17719].” Additionally, Figure 7 legend has been corrected so that “fr, frontal process” is now “fp, frontal process”.

